# Discrepant alterations in main candidate genes among multiple primary melanomas

**DOI:** 10.1186/1479-5876-12-117

**Published:** 2014-05-08

**Authors:** Maria Colombino, MariaCristina Sini, Amelia Lissia, Vincenzo De Giorgi, Ignazio Stanganelli, Fabrizio Ayala, Daniela Massi, Corrado Rubino, Antonella Manca, Panagiotis Paliogiannis, Susanna Rossari, Serena Magi, Laura Mazzoni, Gerardo Botti, Mariaelena Capone, Marco Palla, Paolo A Ascierto, Antonio Cossu, Giuseppe Palmieri

**Affiliations:** 1 Unit of Cancer Genetics, Institute of Biomolecular Chemistry (ICB), National Research Council (CNR) - Traversa La Crucca 3, Baldinca Li Punti, 07100 Sassari, Italy; 2 Dipartimento di Scienze Chirurgiche, Microchirurgiche e Mediche, University of Sassari, Sassari, Italy; 3 Department of Dermatology, University of Firenze, Firenze, Italy; 4 Skin Cancer Unit, Istituto Scientifico Romagnolo per Studio e Cura dei Tumori, Meldola, Italy; 5 Istituto Nazionale Tumori Fondazione Pascale, Napoli, Italy; 6 Department of Pathology, University of Firenze, Firenze, Italy; 7 Unit of Plastic Surgery, University of Salerno, Salerno, Italy

**Keywords:** Multiple melanoma, Mutation analysis, Gene amplification, Melanomagenesis, Molecular classification

## Abstract

**Background:**

Alterations in key-regulator genes of disease pathogenesis (*BRAF*, *cKIT*, *CyclinD1*) have been evaluated in patients with multiple primary melanoma (MPM).

**Methods:**

One hundred twelve MPM patients (96 cases with two primary melanomas, 15 with three, and 1 with four) were included into the study. Paired synchronous/asynchronous MPM tissues (N = 229) were analyzed for *BRAF* mutations and *cKIT*/*CyclynD1* gene amplifications.

**Results:**

*BRAF* mutations were identified in 109/229 (48%) primary melanomas, whereas *cKIT* and *CyclinD1* amplifications were observed in 10/216 (5%) and 29/214 (14%) tumor tissues, respectively. While frequency rates of *BRAF* mutations were quite identical across the different MPM lesions, a significant increase of *cKIT* (p < 0.001) and *CyclinD1* (p = 0.002) amplification rates was observed between first and subsequent primary melanomas. Among the 107 patients with paired melanoma samples, 53 (49.5%) presented consistent alteration patterns between first and subsequent primary tumors. About one third (40/122; 32.8%) of subsequent melanomas presented a discrepant pattern of *BRAF* mutations as compared to incident primary tumors.

**Conclusions:**

The low consistency in somatic mutation patterns among MPM lesions from same patients provides further evidence that melanomagenesis is heterogeneous and different cell types may be involved. This may have implications in clinical practice due to the difficulties in molecularly classifying patients with discrepant primary melanomas.

## Introduction

Incidence of cutaneous melanoma has increased during last decades in Western population [1,2]. Several risk factors have been reported. A light phototype (especially when associated with excessive sun exposure and/or increased incidence of sunburns), a large number of acquired common nevi, and the occurrence of atypical nevi have been associated with a higher risk of melanoma [3,4]. Among others, family history of melanoma (presence of two or, mainly, three or more affected relatives) confers the highest risk for the development of the disease [3,5]. Nevertheless, patients with cutaneous melanoma present a higher incidence of second or even additional melanomas (risk seems to be highest in the first years after diagnosis of the first melanoma and decreases progressively with time) [6,7]. However, subsequent primary melanomas have been found to be significantly thinner than index lesions [8], possibly due to increased surveillance and not to differences in tumor biology [9-11]. In patients with multiple primary melanoma (MPM), the disease staging is based on the melanoma with the worst prognostic features [12].

From the pathogenetic point of view, the *mitogen-activated protein kinase* (MAPK) signal transduction pathway (including the cascade of NRAS, BRAF, MEK1/2, and ERK1/2 proteins) has been reported to play a major role in both the development and progression of melanoma [13,14]. The increased activity of ERK1/2 proteins, which is constitutively activated in melanomas mostly as a consequence of mutations in upstream components of the pathway, has been implicated in rapid melanoma cell growth, enhanced cell survival and resistance to apoptosis [15,16]. Oncogenic mutations of *BRAF*, all constituted by single amino acid substitutions, have been found in approximately 8% of all types of human cancer, including colorectal, ovarian, thyroid, and lung cancers as well as in cholangiocarcinoma and hepatocellular carcinoma [15,17,18], but their highest rates remain those observed in melanoma. Overall, slightly less than half of melanomas carry activating mutations in the *BRAF* gene [19,20], regardless of the mutation screening approach used [21]. The affirmation of new drugs inhibiting some mediators of the MAPK pathway, including mutated BRAF and activated MEK, has led to major advances in the treatment of patients with melanoma [22].

A less common primary pathway which stimulates cell proliferation, without MAPK activation, seems to be the reduction of RB (retinoblastoma protein family) activity by *CyclinD1* or *CDK4* amplification or RB mutation (impaired RB activity through increased CDK4/cyclin D1 could substitute for the MAPK activation and initiate clonal expansion) [23]. Nevertheless, impairment of the p16^CDKN2A^ protein, which acts as an inhibitor of melanocytic proliferation by binding the CDK4/6 kinases and blocking phosphorylation of the RB protein, may also lead to uncontrolled cell growth as well as to increased aggressiveness of transformed melanocytic cells [23,24].

It has been reported that melanomas on skin not chronically exposed to sun usually carry a mutated *BRAF* whereas those arising from chronically sun-damaged (CSD) skin infrequently have *BRAF* mutations but present an increased copy number of the proliferation-controlling *CyclinD1 (CCND1)* or *cKIT* genes, with subsequent increased expression of the correspondent proteins [25-28]. Overexpression of the *CyclinD1* gene is commonly observed in several human cancers, including breast, head and neck, and bladder cancers [29]. In melanoma, the elevated intracellular concentration of *CyclinD1*, related to the amplification of the gene locus at chromosomal level, has been implicated into the resistance to both BRAF and MEK inhibitors since it promotes a MAPK-independent cell proliferation [27,30]. With no stratification for anatomical location, amplification of *cKIT* has been reported in about 7% of all cutaneous melanomas [25,31]; its frequency increase up to 30% or more in acral and CSD melanomas as well as in melanomas carrying a *cKIT* mutation (prevalence is even higher in Chinese population [32]) [25,31,33].

In this study, we aimed at assessing the frequency and distribution of alterations in candidate genes (*BRAF*, *cKIT*, *CyclinD1*) involved in pathogenesis of melanoma in a large series of patients with synchronous or asynchronous MPM lesions.

## Methods

### Patients

One-hundred twelve patients with histologically-proven diagnosis of multiple melanoma (96 cases with two primary melanomas, 15 with three, and 1 with four) were included into the study. Among them, 229 tissue samples of synchronous (N = 40; 17%) or asynchronous (N = 189; 83%) primary melanomas (93 cases with two paired tumor tissues, 13 with three, and 1 case with 4) were available and addressed to somatic molecular analysis. Melanomas were considered as synchronous when a second melanoma was diagnosed during the same first observation or, at the most, within one month from the first diagnosis, as previously stated [34,35]. Among the 189 patients with asynchronous multiple tumors, the subsequent melanomas were diagnosed at a median time from the first diagnosis of 34 months (range, 6-173 months). In particular, intervals between the first diagnosis and the subsequent melanomas were: ≤ 2 years (84 cases; 44%), > 2 to ≤ 4 years (37; 20%), > 4 to ≤ 6 years (34; 18%), > 6 to ≤ 8 years (13; 7%), > 8 to ≤ 10 years (7; 4%), and > 10 years (14; 7%).

Patients were enrolled consecutively between January 2009 and October 2012 from centers in Italy, after evaluation of a collection of 1893 patients with diagnosis of cutaneous melanoma (our series of 112 MPM patients thus represents the 5.9% of the total amount of screened cases). To avoid bias, patients were included regardless of age of onset, cancer family history, and disease characteristics. Familial recurrence of melanoma was ascertained by using a questionnaire to interview patients about their first- and second-degree relatives. Melanoma families were identified according to standardized criteria [36].

Patients were informed about aims and limits of the study and a written consent was obtained for tissue sampling. The study was approved by the ethical review board at the University of Sassari.

### Samples

Paired samples of incident primary melanomas and synchronous or asynchronous subsequent primary melanomas from the same patient were collected. Paraffin-embedded tumor tissues were taken from pathological archives. Using light microscopy, the neoplastic portion of each tissue section was isolated in order to obtain tumor samples with at least 80% neoplastic cells (improving sensitivity of nucleotide sequencing, which may detect a mutation when the mutant alleles are at least 15%-20% of the analyzed DNA sample). Histologic classification and disease stage at diagnosis were confirmed by medical records, pathology reports, and/or review of pathologic material.

### Molecular analysis

For mutation analysis, genomic DNA was isolated from tumor tissues, using standard methods. The coding sequence and splice junctions of the exon 15 in *BRAF* gene were screened by directly sequencing the amplified PCR products, using an automated fluorescence-cycle sequencer (ABIPRISM 3130, Life Technologies, CA). Sequencing analysis was conducted in duplicate (two PCR assays from two different tumor sections) and in both directions (forward and reverse) for all samples. A nucleotide sequence was considered as valid when the quality value (QV) was higher than 20 (<1/100 error probability); in this study, the QV average was 40 (range, 30-45; <1/1000-1/10,000 error probability).

For fluorescence *in situ* hybridization (FISH) analysis, probes specific for *CyclinD1* and *cKIT* genes or control centromeres were labelled with Spectrum Orange or Green (Vysis, Des Plaines, IL), respectively. Three distinct experiments were performed for each case. To be sure that FISH results were exclusively from tumor cells, histologic examination using conventional hematoxylin-eosin staining was systematically carried out on adjacent sections from paraffin-embedded tissues. Digital images were captured using an Olympus BX-61 epifluorescence microscope equipped with the appropriate filters for excitation of DAPI, Cy3 (orange) or FluorX (green), and with a COHU video and Cytovision software. Hybridization signals on at least 200 intact, well-preserved, and non-overlapping nuclei were evaluated by at least two investigators. The *CyclinD1* or *cKIT* gene amplification was defined by the presence of at least a tetrasomic signal (≥2.0 gene copies per control centromere) in more than one tenth (>10%) of cells.

### Statistical analysis

Univariate analysis of the presence of *BRAF*, *CyclinD1,* or *cKIT* alterations versus the various clinical characteristics of the multiple primary melanomas was performed by Pearson’s Chi-Square test, using the statistical package SPSS/7.5 for Windows.

## Results

### Patients and samples

**Figure 1:**
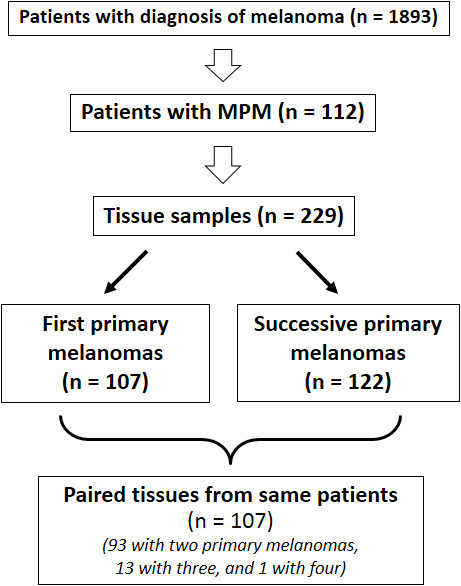
Patients and samples included into the study.

A total of 112 patients with multiple primary melanoma (96 cases with two primary tumors, 15 with three, and 1 with four) were enrolled. Paired samples of synchronous or asynchronous primary melanomas (N = 229; 93 cases with two paired tumor tissues, 13 with three, 1 with four) underwent molecular analysis at somatic level. Overall, a total of 341 samples were screened for mutations in candidate genes, as summarized in Figure 1. Median age of the 112 enrolled patients was 59 years (range, 23-87 years); 59 (53%) were women. Considering the 102 first primary melanomas, trunk was the most frequent location (trunk, 57 [51%]; limbs, 41 [37%]; head and neck, 14 [12%]); median Breslow thickness was 1.7 mm (range, 0.35-5.8 mm).

### Somatic alteration frequencies

*BRAF* mutations were detected in 109 (47.6%) of 229 primary melanomas. All *BRAF* mutations across samples were located in codon 600 of the gene and were of two subtypes only: V600E (94/109; 86.2%) and V600K (15/109; 13.8%) (Table 1). Both mutations are reported in the Human Gene Mutation Database at http://www.hgmd.cf.ac.uk/ac/index.php and the Catalogue Of Somatic Mutations In Cancer (COSMIC) at http://www.sanger.ac.uk/genetics/CGP/cosmic/.

**Table 1:** ***BRAF *** **mutations in 229 tumor tissues from MPM patients**

**Position**	**DNA mutation**	**Amino acid mutation**	**Positive cases n (%)**
Exon 15	c.1799 T > A	p.V600E	94 (41.0)
Exon 15	c.1798_1799GT > AA	p.V600K	15 (6.6)

No association between *BRAF* mutations and any clinicopathological parameters was observed (Table 2). Frequency rates of *BRAF* mutations were quite identical across the different types of MPM lesions (first *vs*. second *vs*. subsequent melanoma; Table 3).

**Table 2:** **Frequency of *BRAF *** **somatic mutations according to patients’ characteristics**

**Subgroups**	**(No. of samples)**	**Cases positive to *BRAF *** **mutations**		
		**No.**	***%***	**P**
**All samples**	(229)	109	47.6	
**Sex**				
Male	(107)	50	*46.7*	0.809
Female	(122)	59	*48.4*	
**Site of primary melanoma**				
Head-neck	(20)	9	*45.0*	0.623
Trunk	(129)	58	*44.9*	
Limbs	(86)	42	*48.8*	
**Number of primary tumors**				
2 melanomas (186)		86	*46.2*	0.172
≥ 3 melanomas (43)		23	*53.5*	
**Type of melanoma**				
Synchronous	(40)	18	*45.0*	0.208
Asynchronous	(189)	91	*48.1*	
**AJCC disease stage**				
≤ I	(161)	72	*44.7*	0.276
≥ II	(78)	37	*47.4*	
**Age at diagnosis**				
< 40 years	(34)	18	*52.9*	0.089
40-50 years	(52)	25	*48.1*	
> 50 years	(143)	66	*46.2*	
**Family history of melanoma**				
1 affected members	(193)	92	47.7	0.962
≥ 2 affected members	(36)	17	47.2	

Disease stage was defined according to the recent American Joint Committee on Cancer (AJCC) guidelines. P: chi-squared test; two tailed; 95% confidence interval.

**Table 3:** Distribution of somatic alterations in multiple melanomas from our series

**Sample**	**Frequency of alterations, positive/total samples (%)**		
	***BRAF *** **mutation**	***cKIT *** **amplification**	***CyclinD1 *** **amplification**
*All melanomas*	*109/229 (47.6)*	*10/216 (4.6)*	*29/214 (13.6)*
First melanoma	50/107 (46.7)	2/101 (2.0)	7/100 (7.0)
Second melanoma	52/107 (48.6)	6/101 (5.9)	21/100 (21.0)
Third/Fourth melanoma	7/15 (46.7)	2/14 (14.3)	1/14 (7.1)

Paraffin-embedded nuclei from available tissue sections of primary melanomas were investigated by a two-colour FISH analysis, using genomic subclones corresponding to either *CyclinD1* or *cKIT* gene loci as well as to the relative chromosome centromeres as controls. Gene amplification, as inferred by the presence of a tetrasomic signal in more than one tenth of cells (see Methods), were observed in cancer cells only. No karyotypic alteration was found in cells from normal tissues surrounding the tumours (diploid signals were consistently detected).

Overall, 10/216 (4.6%) and 29/214 (13.6%) primary melanomas were found to carry *cKIT* and/or *CyclinD1* gene amplification, respectively. As shown in Table 3, a significant increase of *cKIT* amplification rates was observed moving from first to subsequent primary melanomas (p < 0.001); analogously, the rate of *CyclinD1* amplification was significantly higher in subsequent melanomas (22/114; 19.3%) than first primary melanomas (7/100; 7%) (p = 0.002). Again, no correlation between *CyclinD1* or *cKIT* amplification status and any clinicopathological parameters was found (not shown).

Distribution of somatic alterations into the three candidate genes is summarized in Table 4. Among the 229 multiple melanomas analyzed, majority of them (127; 55.5%) presented a genetic alteration in at least one of such genes; no sample was found to carry all three genes affected.

**Table 4:** Somatic alterations in 229 tumor tissues from patients with multiple melanoma

**Alteration type**	**No. of samples**	***%***
*BRAF* mutation only	91	*39.7*
*cKIT* amplification only	6	*2.6*
*CyclinD1* amplification only	9	*3.9*
*BRAF* mutation + *CyclinD1* amplification	17	*7.4*
*BRAF* mutation + *cKIT* amplification	1	*0.4*
*cKIT* + *CyclinD1* amplifications	3	*1.3*
All three genes wild-type	102	*44.5*

Considering the 107 patients who had paired samples of primary melanomas, about half of them showed consistent alteration patterns between either first and second primary tumors (53; 49.5%) or first and third/fourth primary tumors (7/15; 46.7%) (Table 5). Focusing on *BRAF* mutations only, about one third of patients presented discrepant mutation patterns between first and second primary melanomas (34/107; 31.8%); such a discrepancy was even higher when comparing first and third or fourth primary tumors (6/15; 40%) (Table 5). Since differences in genetic alterations underlying melanoma pathogenesis may depend on the anatomical site of the primary lesion [18,25], consistency was evaluated among multiple melanomas arisen into the same body district. Among the 48 (42.9%) patients satisfying such a criterion, again roughly half of them (25; 52.1%) presented consistency in all somatic alteration patterns as well as about one third of cases (17; 35.4%) showed discrepant distribution of *BRAF* mutations (Table 5). No difference in consistency rates was observed between the two subsets of synchronous and asynchronous multiple melanomas (Table 5).

**Table 5:** **Consistency between *BRAF*/*cKIT*/*CyclinD1 *** **alterations in paired samples from patients with multiple melanoma**

**Tissue type**	**No. of cases**	**Cases with consistent mutation patterns, n (%)**	
		***BRAF*** **+** ***cKIT*** **+** ***CyclinD1 *** **alterations**	***BRAF *** **mutations**
*Subsequent vs. first primary melanoma*			
**Second melanoma**	107	53 *(49.5)*	73 *(68.2)*
**Third/Fourth melanoma**	15	7 *(46.7)*	9 *(60.0)*
*Subsequent vs. second primary melanoma*			
**Third/Fourth melanoma**	15	8 *(53.3)*	11 *(73.3)*
*Multiple melanomas at the same anatomical site (head/neck - trunk - limbs)*			
**All cases**	48	25 *(52.1)*	31 (*64.6*)
Synchronous	13	7 *(53.8)*	8 (*61.5*)
Asynchronous	35	18 *(51.4)*	23 *(65.7)*

Among the 62 paired samples (54/107 [50.5%] patients) with discrepancies in *BRAF/cKIT/CyclinD1* mutation patterns between first and subsequent primary melanomas, majority of them (40; 64.5%) displayed differences in *BRAF* mutation distribution (19 with a wild-type first tumor and a mutated subsequent tumor, 19 with a mutated first tumor and a wild-type subsequent tumor, and 2 with a change in mutation variants between the two tumor lesions) (Additional file 1: Table S1). The remaining 22 (35.5%) discrepant paired samples showed differences in *cKIT* and/or *CyclinD1* gene amplification status (Additional file 1: Table S1). A quite similar distribution of genetic alterations into the three candidate genes was observed when comparing subsequent *versus* second primary melanomas (Additional file 2: Table S2).

The *BRAF/cKIT/CyclinD1* mutation status was not evaluated for association with clinical outcome in our series.

## Discussion

Melanoma development and progression have been reported to occur by sequential accumulation of genetic and molecular alterations [18,37]. Two main genetic networks have been demonstrated to play a crucial role in the control of growth, proliferation, and survival of the melanocyte cells: the CDKN2A-driven pathway and the *mitogen-activated protein kinase* (MAPK) signal transduction cascade [38,39]. Genetic alterations in different members of these pathways have been associated with the pathogenesis of distinct types of primary melanomas: high frequency of *BRAF* or *NRAS* mutations (which are mutually exclusive) is mostly frequent in melanoma on skin without chronic sun-damage, whereas *CyclinD1* or *cKIT* amplifications are prevalent in CSD or acral melanoma, respectively. In our study, we investigated the prevalence and distribution of such genetic alterations in MPM patients.

A high prevalence of somatic mutations in *BRAF* gene was detected in incident and subsequent melanomas. The frequency of *BRAF* mutations in primary melanomas (47%) was consistent with that observed in our previous study on 451 Italian patients with single melanoma (49%) [40] and slightly higher than that reported in a meta-analysis on 2521 patients with cutaneous melanomas (41%) [41]. In our series, two BRAF^V600^ mutation subtypes were detected: V600E and V600K (in 41% and 7% of cases, respectively). Such two variants represent the most prevalent *BRAF* mutations (our frequencies were consistent with most of those reported in literature [41]) and are able to constitutively activate BRAF kinase [21]. Amplification of *CyclinD1* and *cKIT* genes, as determined by FISH analysis, was found in about 14% and 5% of melanoma tissues from our series, respectively (see Table 3). Again, such frequencies were consistent with those reported in literature (ranging from 12% to 19% for *CyclinD1* amplification [27,42-44] and calculated in about 7% of all cutaneous melanomas for *cKIT* amplification [25,31]). One (0.4%) out of 229 melanoma samples presented a coexistence of *BRAF* mutation and *cKIT* amplification (see Table 4), confirming that aberrations in these two genes can be considered as mutually exclusive [26].

A markedly higher rate of either *BRAF* mutations (59%) or *CyclinD1* (38%) or *cKIT* (13%) amplifications was previously observed in 32 melanoma cell lines as controls by our group ([45] and unpublished data). As reported [45], these control cell lines were established as primary cell cultures from tumor samples obtained from donor patients with documented diagnosis of melanoma. Since cultured melanomas are thought to represent cells with the most malignant phenotype, one could speculate that genetic alterations in these three candidate genes play a role in tumor progression.

Sixty-two paired samples from 54 (51%) patients showed discrepancies in *BRAF/cKIT/CyclinD1* mutation patterns between first and subsequent primary melanomas (see Table 5). In the discrepant cases, we observed 20 (37%) patients with a wild-type first tumor and a mutated subsequent tumor, 14 (26%) with a mutated first tumor and a wild-type subsequent tumor, 8 (15%) with change in alteration variants between the two tumor lesions, and 12 (22%) with an additional gene amplification in the two BRAF-mutated tumors (3 cases in first but not in subsequent tumors and 9 with an opposite condition). In majority of cases (29/54; 53%), gene alterations seem to be acquired in subsequent melanomas. Moreover, while *BRAF* mutations were equally distributed among discrepant multiple melanomas (47.5% wild-type first tumors and mutated subsequent tumors, 47.5% mutated first tumor and wild-type subsequent tumors), rates of *cKIT* and *CyclinD1* amplification were found to significantly increase moving from incident to subsequent primary melanomas (p values, <0.001 and 0.002, respectively). Such discrepancies were also confirmed among paired primary melanomas located at the same anatomical site as well as in synchronous primary melanomas (see Table 5). Overall, these observations provide evidence about the heterogeneity of the molecular mechanisms underlying the development of MPM in the same patients. The knowledge that molecularly heterogeneous cell types may coexist in primary melanomas [45,46] is a further confirmation that complex pathogenetic scenarios exist in melanomagenesis.

About one third of patients presented a discrepant pattern of *BRAF* mutations between incident and subsequent primary melanomas (overall, 40/122; 32.8%). The introduction into the clinical practice of vemurafenib and dabrafenib, potent inhibitors of BRAF^V600^ mutants, makes the assessment of *BRAF* mutations as a crucial step toward the appropriate use of a targeted melanoma treatment. The low consistency in *BRAF* mutation patterns among MPM lesions from the same patients arises the practical question on how cases with coexistence of *BRAF*^*wild-type*^ and *BRAF*^*mutant*^ primary melanomas (and, to a less extent, those carrying different *BRAF* variants - which may present a different degree of responsiveness to BRAF inhibitors) should be molecularly classified. Nevertheless, progression of disease in patients with such discrepancies in primary melanomas may suggest taking into consideration all developing metastases for *BRAF* mutation analysis cucaccording to the recent indications provided by the National Comprehensive Cancer Network (NCCN; at http://www.nccn.org/professionals/physician_gls/f_guidelines.asp) guidelines, most recent melanoma tissue samples should be considered as adequate for *BRAF* mutation screening].

In our study, we contributed to provide additional clues about the prevalence of alterations in some candidate genes (with particular attention to *BRAF* mutations) among synchronous or asynchronous multiple primary melanomas. Our findings further support evidence that molecular events underlying development and progression of melanoma are really complex. A better comprehension of the factors crucially involved in activating one or the other pathogenetic molecular mechanism, even in the same individual, might have an impact on the disease management. Since the future of melanoma therapy is likely to focus on targeting multiple pathways, advancing technologies (i.e., deep-sequencing approaches) will permit to simultaneously investigate multiple genes and targets toward more accurate correlations between molecular signatures and clinical outcome.

## Abbreviations

MPM: Multiple primary melanoma; MAPK: Mitogen-activated protein kinase; PCR: Polymerase chain reaction; FISH: Fluorescence *in situ* hybridization.

## Competing interests

PAA is consultant of Bristol Myers Squibb, MSD, and Roche-Genentech. He participated into the Advisory Board from Bristol Myers Squibb, MSD, Roche-Genentech, GSK, Amgen, Celgene, Medimmune, and Novartis. He received honoraria from Brystol Myers Squibb, MSD, and Roche-Genentech. All remaining authors declare the absence of any Competing Interest.

## Authors’ contributions

MCo, performed mutation analysis and data interpretation, helped to draft the manuscript; MCS, performed FISH analysis and data interpretation; AL, performed quality control of pathological data; VDG, IS, FA, DM, CR, SR, SM, LM, GB, MP, and PAA participated in patients’ collection and data acquisition; AM, PP, and MCa, performed data analysis; AC, performed pathological review and participated into the design of the study; GP, performed data interpretation, conceived of the study, drafted the manuscript. All authors read and approved the final manuscript.

## Supplementary Material

Additional file 1: Table S1 Mutation patterns in patients presenting discrepancies in tumor lesions (54 second and 8 third/fourth vs. first primary melanomas) for *BRAF*/*cKIT*/*CyclinD1* alterations. Click here for file

Additional file 2: Table S2 Mutation patterns in patients presenting discrepancies in tumor lesions (7 subsequent *vs*. second primary melanomas) for *BRAF*/*cKIT*/*CyclinD1* alterations. Click here for file
