# Supplementary material for: Discrepant alterations in main candidate genes among multiple primary melanomas
Source: J Transl Med. 2014 May 8;12:117. doi: 10.1186/1479-5876-12-117 (PMC4023698; doi:10.1186/1479-5876-12-117)
Supplement: Additional file 1: Table S1 — Mutation patterns in patients presenting discrepancies in tumor lesions (54 second and 8 third/fourth vs. first primary melanomas) for BRAF/cKIT/CyclinD1 alterations. [file 1479-5876-12-117-S1.doc]

***Additional file 1: Table S1.*** *Mutation patterns in patients presenting discrepancies in tumor lesions (54 second and 8 third/fourth vs. first primary melanomas) for BRAF/cKIT/CyclinD1 alterations.*

| **Tissue type** | **Mutation patterns among discrepant paired samples** | | | | | |
| --- | --- | --- | --- | --- | --- | --- |
|  | ***BRAF*** | | ***cKIT*** | | ***CyclinD1*** | |
|  | **First tumor** | **Subsequent tumor** | **First tumor** | **Subsequent tumor** | **First tumor** | **Subsequent tumor** |
| **Second melanoma** | wt  wt  V600E  wt  wt  V600E  wt  V600E  V600E  wt  V600E  wt  wt  V600E  V600E  wt  V600E  wt  wt  V600E  V600E  V600E  wt  wt  wt  V600K  V600E  wt  wt  wt  V600E  wt  wt  V600E  wt  V600E  V600K  wt  V600E  V600E  V600E  wt  V600K  V600E  wt  V600E  wt  wt  V600K  wt  V600E  V600E  wt  V600K | V600E  wt  wt  V600E  V600E  wt  wt  V600E  wt  V600E  V600E  wt  wt  wt  wt  V600E  wt  V600E  V600E  wt  wt  V600K  V600E  V600E  wt  wt  wt  wt  V600E  wt  wt  V600E  wt  V600E  V600K  V600E  wt  V600E  V600E  wt  V600E  V600E  V600K  V600E  V600K  V600E  wt  V600E  V600K  V600E  V600E  wt  V600E  wt | dis  dis  dis  dis  dis  dis  dis  dis  dis  dis  dis  AMPL  dis  dis  dis  dis  dis  dis  dis  dis  dis  dis  dis  dis  dis  dis  dis  dis  dis  dis  dis  dis  AMPL  dis  dis  dis  dis  dis  dis  dis  dis  dis  dis  dis  dis  dis  AMPL  dis  dis  dis  dis  dis  dis  dis | dis  dis  dis  dis  dis  dis  dis  dis  dis  dis  dis  dis  dis  dis  dis  dis  dis  dis  dis  AMPL  dis  dis  dis  dis  AMPL  dis  dis  AMPL  dis  AMPL  dis  dis  dis  dis  dis  dis  dis  dis  dis  dis  AMPL  dis  dis  dis  dis  dis  dis  dis  dis  dis  dis  dis  dis  dis | dis  AMPL  dis  dis  dis  dis  dis  AMPL  dis  dis  dis  dis  dis  dis  dis  dis  dis  AMPL  AMPL  dis  dis  dis  dis  dis  dis  dis  dis  dis  dis  dis  dis  dis  dis  dis  dis  dis  dis  dis  AMPL  dis  dis  dis  dis  dis  dis  dis  dis  dis  dis  dis  dis  dis  AMPL  dis | dis  dis  dis  dis  dis  AMPL  AMPL  dis  AMPL  AMPL  AMPL  dis  AMPL  dis  dis  AMPL  dis  dis  dis  AMPL  AMPL  dis  AMPL  dis  AMPL  dis  dis  dis  AMPL  dis  dis  dis  dis  AMPL  dis  AMPL  dis  dis  dis  dis  dis  dis  AMPL  AMPL  dis  AMPL  dis  dis  AMPL  dis  AMPL  dis  AMPL  dis |
| **Third/ Fourth melanoma** | V600E  V600E  wt  V600E  wt  V600E  V600E  V600K | wt  V600E  wt  wt  V600E  wt  wt  V600E | dis  dis  dis  dis  AMPL  dis  dis  dis | dis  dis  AMPL  dis  dis  dis  dis  dis | dis  dis  dis  dis  dis  dis  AMPL  dis | dis  AMPL  AMPL  dis  dis  dis  dis  dis |

*dis, disomy; AMPL, gene amplification, wt, wild-type*
