# Supplementary material for: Discrepant alterations in main candidate genes among multiple primary melanomas
Source: J Transl Med. 2014 May 8;12:117. doi: 10.1186/1479-5876-12-117 (PMC4023698; doi:10.1186/1479-5876-12-117)
Supplement: Additional file 2: Table S2 — Mutation patterns in patients presenting discrepancies in tumor lesions (7 subsequent vs. second primary melanomas) for BRAF/cKIT/CyclinD1 alterations. [file 1479-5876-12-117-S2.doc]

**Additional file 2: Table S2.** Mutation patterns in patients presenting discrepancies in tumor lesions (7 subsequent *vs*. second primary melanomas) for *BRAF*/*cKIT*/*CyclinD1* alterations

| **Tissue type** | **Mutation patterns among discrepant paired samples** | | | | | |
| --- | --- | --- | --- | --- | --- | --- |
|  | ***BRAF*** | | ***cKIT*** | | ***CyclinD1*** | |
|  | **Second tumor** | **Subsequent tumor** | **Second tumor** | **Subsequent tumor** | **Second tumor** | **Subsequent tumor** |
| **Third/ Fourth melanoma** | wt  V600E  V600E  wt  wt  V600E  wt | V600E  V600E  wt  wt  wt  wt  V600E | dis  dis  dis  dis  AMPL  dis  dis | dis  dis  dis  AMPL  dis  AMPL  dis | dis  dis  dis  dis  dis  dis  dis | dis  AMPL  dis  dis  dis  dis  dis |

*dis, disomy; AMPL, gene amplification, wt, wild-type*
